# Agmatine Reduces Lipopolysaccharide-Mediated Oxidant Response via Activating PI3K/Akt Pathway and Up-Regulating Nrf2 and HO-1 Expression in Macrophages

**DOI:** 10.1371/journal.pone.0163634

**Published:** 2016-09-29

**Authors:** Jianshen Chai, Li Luo, Fengyan Hou, Xia Fan, Jing Yu, Wei Ma, Wangqi Tang, Xue Yang, Junyu Zhu, Wenyuan Kang, Jun Yan, Huaping Liang

**Affiliations:** 1 State Key Laboratory of Trauma, Burns and Combined Injury, Research Institute of Surgery, Daping Hospital, Third Military Medical University, Chongqing 400042, China; 2 School of Life Science and Engineering, Southwest Jiaotong University, Chengdu 610031, Sichuan, China; University of PECS Medical School, HUNGARY

## Abstract

Macrophages are key responders of inflammation and are closely related with oxidative stress. Activated macrophages can enhance oxygen depletion, which causes an overproduction of reactive oxygen species (ROS) and leads to further excessive inflammatory response and tissue damage. Agmatine, an endogenous metabolite of L-arginine, has recently been shown to have neuroprotective effects based on its antioxidant properties. However, the antioxidant effects of agmatine in peripheral tissues and cells, especially macrophages, remain unclear. In this study we explored the role of agmatine in mediating antioxidant effects in RAW 264.7 cells and studied its antioxidant mechanism. Our data demonstrate that agmatine is an activator of Nrf2 signaling that markedly enhances Nrf2 nuclear translocation, increases nuclear Nrf2 protein level, up-regulates the expression of the Nrf2 downstream effector HO-1, and attenuates ROS generation induced by Lipopolysaccharide (LPS). We further demonstrated that the agmatine-induced activation of Nrf2 is likely through the PI3K/Akt pathway. LY294002, a specific PI3K/Akt inhibitor, abolished agmatine-induced HO-1 up-regulation and ROS suppression significantly. Inhibiting HO-1 pathway significantly attenuated the antioxidant effect of agmatine which the products of HO-1 enzymatic activity contributed to. Furthermore, the common membrane receptors of agmatine were evaluated, revealing that α2-adrenoceptor, I1-imidazoline receptor or I2-imidazoline receptor are not required by the antioxidant properties of agmatine. Taken together, our findings revealed that agmatine has antioxidant activity against LPS-induced ROS accumulation in RAW 264.7 cells involving HO-1 expression induced by Nrf2 via PI3K/Akt pathway activation.

## Introduction

Lipopolysaccharide (LPS), a major component of bacterial cell walls, can stimulate inflammatory responses and induce reactive oxygen species (ROS) production in various cell types [1]. In immunocytes, such as macrophages, ROS accumulation promotes the overexpression of proinflammatory cytokines, which aggravates inflammatory responses and does harm to local tissue [2]. Correspondingly, inhibiting ROS production in macrophages is regarded as a general way to attenuate proinflammatory signals [3]; therefore, the signaling pathways associated with ROS production and clearance have become the focus of intense research.

The nuclear factor (erythroid 2-derived)-like 2 (Nrf2) is the crucial regulator that counteracts ROS generation by activating antioxidant cascades [2]. Normally, Kelch-like ECH-associated protein 1 (Keap1), the repressor of Nrf2, binds Nrf2 in a complex, sequestering it in the cytoplasm [4, 5]. Upon stimulation by oxidative and electrophilic chemical signals, Nrf2 is released from Keap1, and translocates into the nucleus, where it binds to anti-oxidant responsive elements (ARE) [6–8]. Many cellular protective genes, such as heme oxygenase-1 (HO-1) and NAD(P)H quinine oxidoreductase-1(NQO1) [9, 10], are target genes of nuclear Nrf2. HO-1 is a member of the intracellular phase II enzyme family, which are ubiquitously expressed at low levels in resting conditions, and highly up-regulated by numerous stress stimuli [11], such as LPS and Heme. Several kinases such as phosphatidylinositol 3-kinase (PI3K), c-Jun N-terminal kinase1/2 (JNK1/2), and extracellular signal-regulated kinase 1/2 (ERK1/2) have been confirmed to promote HO-1 expression. Experimental evidence has shown that HO-1 plays an important role in host defense against ROS generation and oxidative injury [12, 13]. It also contributes to the anti-inflammatory activity of cells and tissues [14].

Agmatine, an endogenous polyamine, is the product of the decarboxylation of arginine [15] and is mainly present in the brain as a neurotransmitter. Accumulating evidence has indicated that agmatine is an effective drug to treat depression, and its antidepressant-like effect is suggested to involve controlling pro-/anti-oxidative homeostasis in the hippocampus [16]. Andiara et al. also confirmed that agmatine affords neuroprotection against corticosterone-induced toxicity by a mechanism that involves the Nrf2 pathway and HO-1 expression [17]. Recent studies have shown that agmatine also protects microglia and astrocytes from oxidative stress both *in vitro* and *in vivo* [18, 19]. However, agmatine is not just in the brain but also spreads into other tissues in mammals. The potential antioxidant effects of agmatine in peripheral cells and tissues, especially in macrophages remain unclear. We have previously demonstrated that agmatine inhibited NF-κB signaling in the lungs and protected mice against Zymosan-induced acute lung injury, suggesting that agmatine may be a potential safe and effective approach for the treatment of acute lung injury [20]. However, the anti-inflammatory and antioxidant mechanisms of agmatine in acute lung injury remain unknown.

In this study we explored the mechanism by which agmatine protects RAW 264.7 cells against LPS-induced oxidative stress *in vitro*. We examined the association between agmatine and the Nrf2/HO-1 antioxidant signaling. Our results revealed that the PI3K/Akt pathway participates in agmatine-induced Nrf2 activation and antioxidant activity. Furthermore, we studied the membrane receptors that control this anti-oxidative response.

## Materials and Methods

### Reagents

Agmatine, LPS (Escherichia coli 0111:B4) and protoporphyrinIX zinc(II) (ZnPP)were purchased from Sigma–Aldrich (Saint Louis, MO, USA). LY294002, protease/phosphatase inhibitor cocktail, anti-β-actin, anti-phosphorylated Akt and anti-total Akt antibodies were products of Cell Signaling Technology (Danvers, MA, USA). Idazoxan, anti-Nrf2 and anti-iNOS antibodies were purchased from Santa Cruz Biotechnology (Santa Cruz, CA, USA).Anti-HO-1 antibody was product of Abcam (Cambridge, MA, USA). RPMI 1640 and fetal bovine serum were products of Gibco-BRL Invitrogen (San Diego, CA, USA). Yohimbine, efaroxan, dimethyl sulfoxide (DMSO), 2′,7′-dichlorofluorescin diacetate (DCFH-DA) and 3-(4,5-dimethylthiazol-2-yl)-2,5-diphenyltetrazolium bromide (MTT) were obtained from Sigma-Aldrich. BCA protein assay kit, total nitric oxide assay kit and nuclear and cytoplasmic protein extraction kits were from Beyotime (Jiangsu, China).

### Cell Culture

Mouse RAW 264.7 cells were purchased from the American Type Culture Collection (Rockville, MD, USA). Cells were cultured in RPMI 1640 supplemented with 10% fetal bovine serum, 100 U/mL penicillin, and 100 μg/mL streptomycin at 37°C in a humidified incubator with 5% CO_2_. For stimulation experiments, RAW 264.7 cells were seeded at 1 × 10^6^ cells/well in 6-well culture plates and were stimulated with 10 μg/mL LPS for the indicated time intervals.

### Cell Viability by MTT

RAW 264.7 cells were seeded at a density of 5000 cells/well into 96-well plates, treated with or without agmatine (50000, 10000, 2000, 400, 80, 16, 3.2, 0.64, 0.128 and 0 μM) for 24 h. Subsequently, 10 μL of 5 mg/mL MTT solution was added to form a purple formazan. Afterwards, 100 μL of dimethyl sulfoxide (DMSO) was transferred into each well to dissolve the purple formazan, and results were measured using a microplate reader (Biotek, Winooski, VT, USA) at an absorbance of 570 nm. The IC50 values were obtained from the MTT viability growth curve.

### Nitric Oxide (NO) Detection Assay

The nitrite levels in the culture medium were assessed by Griess reaction. RAW 264.7 cells (2 × 10^5^ cells/well) were cultured in 24-well plates for 24 h, treated with or without LPS (10 μg/mL) co-incubated with agmatine (1 mM, 30 min following LPS treatment). Cellular NO production was stimulated by adding monosodium urate (MSU) crystals (1 mg/mL), followed by incubation for 24 h. The conditioned medium (100 μL) was then mixed with equal volumes of Griess reagent and incubated for 15 min. The absorbance of the mixture at 540 nm was measured with an ELISA microplate reader (Biotek). The values were compared with those from standard concentrations of sodium nitrite and then levels of nitrite in the conditioned media from treated cells were calculated.

### Detection of ROS Generation

The effect of agmatine on the generation of intracellular ROS was estimated using the DCFH-DA probe. Briefly, RAW 264.7 cells, cultured in 24-well plates for 24 h before the experiment, were stimulated with LPS (10 μg/mL) for 30 min to induce ROS production, and then treated with agmatine (1 mM) for 24 h. Cells were washed twice with PBS and then incubated with the DCFH-DA probe (20 μmol/L) at 37°C for 30 min, and then washed immediately with PBS. The images were obtained using a fluorescence microscope. The fluorescence intensity was analyzed by flow cytometry (ACEA NovoCyte, Hangzhou, China). A minimum of 10,000 events/sample were acquired.

### Determination of bilirubin in culture medium

Cells were incubated with 10μg/mL LPS for 24h in the presence or absence of 1 Mm agmatine, and bilirubin was determined in the culture supernatant at the 24^th^ hour. Briefly, 0.5 ml of culture supernatant was added to 250 mg of barium chlorate monohydrate and vortex-mixed thoroughly. Benzene was then added to the mixture and tubes were vigorously vortex-mixed again. The benzenephase containing the extracted bilirubin was separated from the aqueous phase by centrifugation. Bilirubin was measured spectrophotometrically and calculated in picomoles/ml of medium[13].

### Western Blotting

RAW 264.7 cells were stimulated with LPS (10 μg/mL) for 30 min, and then treated with agmatine (1 mM) for 24 h. Cell lysates were prepared using nuclear and cytoplasmic extraction kits according to the manufacturer’s instructions (Beyotime) for the detection of Nrf2 protein. Whole cell lysate was prepared in ice-cold RIPA buffer mixed with protease/phosphatase inhibitor cocktails for the detection of iNOS, Akt and p-Akt protein. Samples were denatured, and equal amounts of protein were subjected to 9% SDS-PAGE, and then transferred into polyvinylidene fluoride membranes. The membranes were blocked with 1–5% bovine serum albumin at room temperature for 1 h and incubated with the indicated primary antibodies, Nrf2 (1:200), anti-HO-1(1:10000), iNOS (1:500), Akt (1:1000), p-Akt (1:1000), GAPDH (1:1000) and PCNA (1:1000) overnight at 4°C. The next day, membranes were washed with Tris-buffered saline solution containing 0.1% Tween 20 (TBST) and probed for 1 h with HRP-conjugated goat anti rabbit IgG antibody (1:10000). The immunoreactive bands were detected using the enhanced chemiluminescence detection system (Bio-Rad Laboratories, Hercules, CA, USA), and band intensities were measured by densitometric analysis using Image J software.

### Quantitative RT-PCR Analysis

Total RNA was extracted using Trizol reagent (Sigma–Aldrich) following the manufacturer’s instructions. Total RNA concentration and purity were determined using a Nanodrop Spectrometer 200c (Thermo Fisher, Boston, MA, USA) and only RNA with an absorbance 260/280 ratio ranging from 1.9 to 2.1 was used for experiments. A 1 μg sample of total RNA was subsequently reverse transcribed for first-strand cDNA synthesis using the PrimeScriptTM RT reagent Kit with gDNA Eraser-Perfect Real Time (TaKaRa, Shiga, Japan) under the following conditions: 37°C for 15 min, 85°C for 5 s, and then maintained at 4°C. QPCR was performed in triplicate on a BioRad CFX96 (Hercules, CA, USA) using 1 μg cDNA template and SYBR Premix Ex TaqTM II kit (TaKaRa) and the primer sequences: IL-6(F) 5´-ACCACGGCCTTCCCTACTTC, (R) 5´-CTCATTTCCACGATTTCCCAG; HO-1(F) 5´-AAGCCGAGAATGCTGAGTTCA, (R) 5´-GCCGTGTAGATATGGTACAAGGA; NQO-1(F) 5´-AGGATGGGAGGTACTCGAATC, (R) 5´-AGGCGTCCTTCCTTATATGCTA; β-actin(F) 5´-AGCCATGTACGTAGCCATCC, (R) 5´-CTCTCAGCTGTGGTGGTGAA. Relative mRNA expression was calculated by the 2–(Ct) method and normalized to β-actin.

### Electrophoretic Mobility Shift Assay (EMSA)

Nuclear proteins from treated RAW 264.7 cells were extracted with Nuclear and Cytoplasmic Extraction kits (Beyotime) and quantified using the BCA assay (Beyotime). To measure the binding capacity of the nuclear proteins and Nrf2 binding domain (ARE), the DNA-protein complexes were resolved on 6.5% mini Gels for 40 min at 180V and detected at 700 nm using the Odyssey scan bed (LiCor, Lincoln, NE, USA). The probe consisted of an oligonucleotide containing the ARE of HO-1 enhancer 5´-TTTTATGCTGTGTCATGGTT-3´.

### Statistical Analysis

Data were presented as mean ± SEM. Differences in each group were tested using one-way ANOVA with SPSS 17.0 software (IBM, Armonk, NY, USA), and differences were accepted as significant when p < 0.05.

## Results

### MTT-Based Cytotoxicity Assay

**Fig 1:**
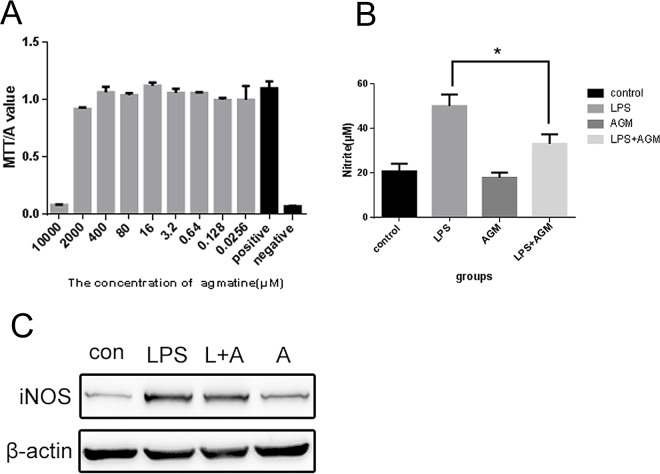
Effects of agmatine on cell viability, NO production and iNOS expression in LPS-induced RAW 264.7 cells. (A) Cell viability; after treatment in 96-well plates with various doses of agmatine for 24 h, the MTT-based cytotoxicity assay was performed. Absorbance value was read at 570 nm. Results are expressed as the mean ± SEM from three independent experiments. Data were analyzed by one-way ANOVA. (B) NO production; RAW264.7 cells, seeded at a density of 2×10^5^ cells/well in 24-well plates for 24 h, were treated with or without LPS (10 μg/mL) and co-incubated with agmatine (1 mM, 30 min following LPS treatment). The culture medium was removed to measure NO according to the Griess protocol. (C) iNOS detection; cells were seeded at a density of 5×10^6^ cells/well into 10 cm plates, treated with LPS and agmatine, and assayed for iNOS expression at 24 h.

RAW 264.7 cells were treated with different concentrations of agmatine (50000, 10000, 2000, 400, 80, 16, 3.2, 0.64, 0.128 and 0 μM) for 24 h and the IC50 value was calculated. As shown in Fig 1A, agmatine produced no effect on cell viability at concentrations below 2 mM. Therefore, 1 mM agmatine was chosen for further experiments.

### Agmatine Reduces the LPS-Induced Increase of NO and iNOS Levels

Nitric oxide (NO), a production of nitric oxide synthase (NOS) family, acts as a biological mediator and plays important roles in LPS-stimulated macrophages. In this study, NO production in LPS-treated cells was evaluated using a colorimetric assay based on the Griess reaction, and the expression of iNOS was measured by western blot. The results show that LPS significantly increased NO production and enhanced iNOS protein levels in RAW 264.7 cells. Agmatine treatment significantly decreased the LPS-induced excess NO production and iNOS overexpression (Fig 1B and 1C).

### Agmatine Therapeutically Reduces LPS-Induced ROS Generation

**Fig 2:**
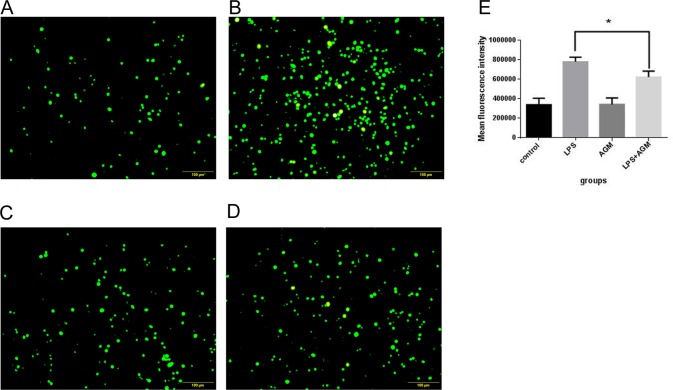
Agmatine therapeutically reduces LPS-induced ROS generation. ROS detection was performed using a fluorescence probe. (A) RAW264.7 cells were cultured in RPMI 1640 for 24 h followed by incubation with the DCFH-DA probe (20 μmol/L) at 37°C for 30 min. (B) RAW264.7 cells were stimulated with 10 μg/mL LPS for 24 h followed by incubation with the DCFH-DA probe for 30 min. (C) RAW264.7 cells were treated with 1 mM agmatine for 24 h. (D) RAW264.7 cells were incubated with 10 μg/mL LPS and 1 mM agmatine added 30 min later for 24 h, then the DCFH-DA probe was used for 30 min. (E) ROS production was analyzed by flow cytometry. A minimum of 10000 events/sample were acquired. Results were means ± SEM of three independent experiments and were assessed by one-way ANOVA. * P < 0.05 indicates significant differences compared with the control group.

Overproduction of ROS is deleterious to normal cells because of its ability to induce overexpression of cytokines in macrophages. To assess the amount of ROS generated by LPS exposure, as well as the ability of agmatine to relieve such effects, RAW 264.7 cells were pretreated with LPS (10 μg/mL) for 30 min, then co-incubated with agmatine (1 mM) for 24 h. ROS production was measured with the molecular probe DCFH-DA and detected by flow cytometry. The results showed that LPS significantly increased the amounts of ROS (Fig 2B); however, agmatine treatment significantly attenuated ROS production in LPS-pretreated cells (Fig 2D). Agmatine alone had no effect on this ROS production (without LPS pretreatment). Both fluorescence staining and assay values demonstrated that agmatine could therapeutically reduce LPS-Induced ROS generation in macrophages (Fig 2E).

### Agmatine Enhances HO-1 Expression and Activates the Transcription Factor Nrf2

**Fig 3:**
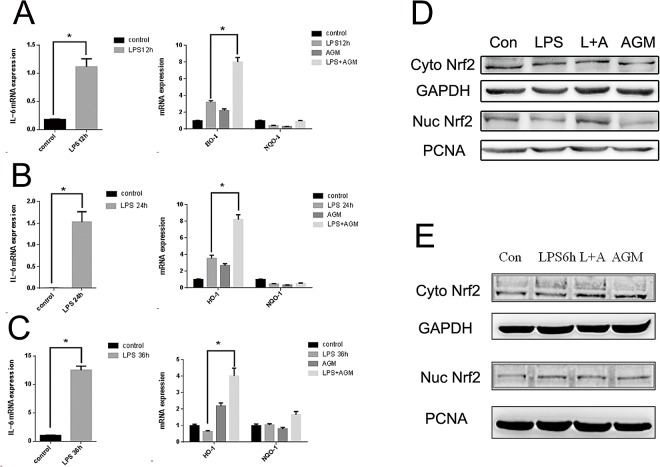
Agmatine induces an up-regulation of HO-1 and activates the transcription factor Nrf2. RAW264.7 cells were incubated with 10 μg/mL LPS for 30min, and then 1 mM agmatine was added into the medium and incubated for additional (A)12h, (B)24h or (C)36h, respectively. Then the mRNA expressions of HO-1 and NQO-1 were analyzed by qPCR. (D and E) RAW264.7 cells were incubated with 10 μg/mL LPS and 1 mM agmatine added 30 min later for 24h(D) and 6 h(E), and the protein expression of Nrf2 was analyzed by western blotting. Results were expressed as mean ± S.E.M (n = 3) and were assessed by one-way ANOVA. * P < 0.05 and ** P < 0.01 indicate significant differences compared with the control group.

**Fig 4:**
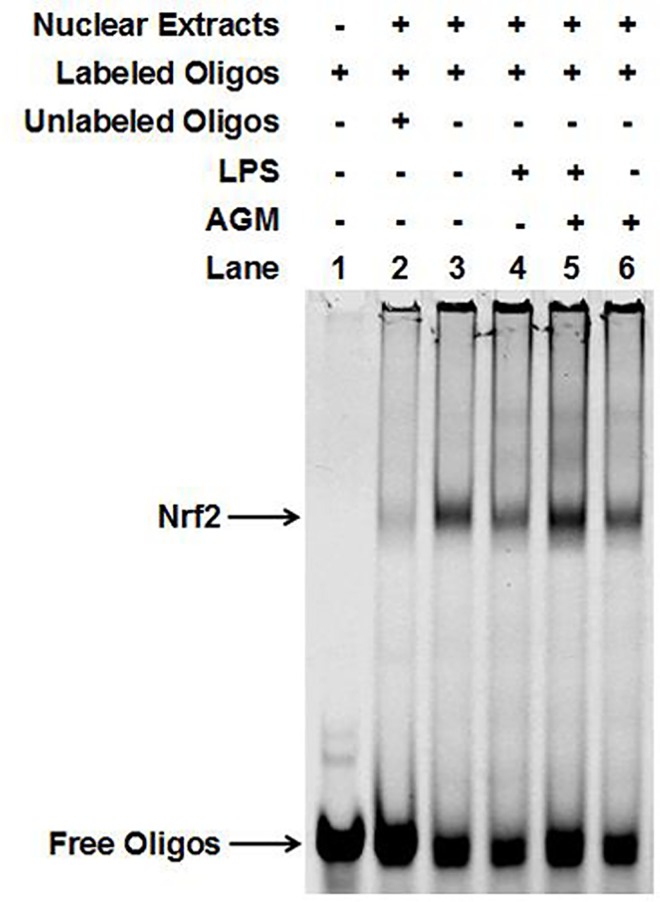
Nrf2 binding assay by EMSA. RAW264.7 cells were incubated with 10 μg/mL LPS and 1 mM agmatine added 30 min later for 24 h. The DNA binding activity of Nrf2 was measured by EMSA as described in the Materials and Methods section.

HO-1 plays an important role in host defense against oxidative injury and is induced by various stress stimuli. To confirm whether agmatine mediates antioxidant activity by up-regulating HO-1 and NQO-1, HO-1 and NQO-1 mRNA expression was detected by qPCRin LPS-induced cells treated with agmatine for different durations (12, 24 and 36 h). The results showed that agmatine significantly increased HO-1 mRNA expression, especially in time of 24 h (Fig 3B). However, NQO-1 levels were unaffected (Fig 3A–3C). Furthermore, to determine whether agmatine up-regulated HO-1 levels by Nrf2 activation, which is a vital signaling mediator in regulating cellular defense against oxidative stress, western blot analysis was performed. The results showed that after 24 h agmatine treatment, nuclear Nrf2 was significantly increased (Fig 3D). However, that was not apparent after a 6h short time reaction (Fig 3E). Additionally, the EMSA assays showed that agmatine increased the DNA binding activity of Nrf2 (Fig 4).

### PI3K/Akt Regulates the Agmatine-Induced Anti-Oxidation Effects in RAW 264.7 Cells

**Fig 5:**
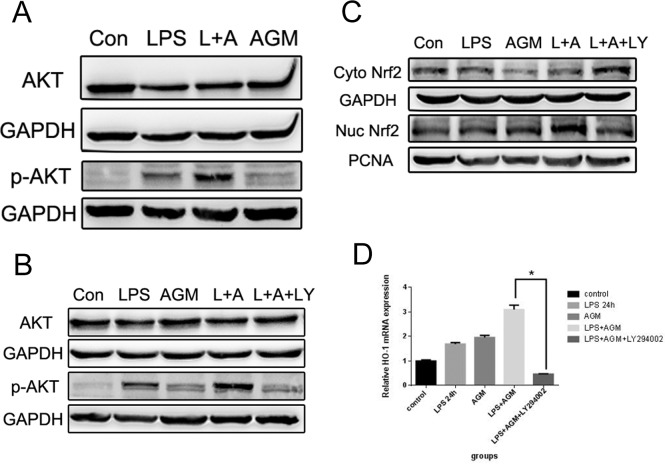
PI3K/Akt signal regulates agmatine-induced Nrf2 activation and HO-1 expression in RAW264.7 Cells. (A) RAW264.7 cells were treated with 1 mM agmatine for 24 h, and total Akt and phospho-Akt (p-Akt) levels were analyzed by western blotting. (B, C) RAW264.7 cells were pretreated for 1 h with 10 μM LY294002, and total Akt, p-Akt and Nrf2 expression were analyzed by western blotting. (D) HO-1 mRNA expression after LY294002 pretreatment were analyzed by qPCR. Results are means ± SEM of three independent experiments and were assessed by one-way ANOVA. *P < 0.05 and **P < 0.01 indicate significant differences compared with the agmatine-treated group.

**Fig 6:**
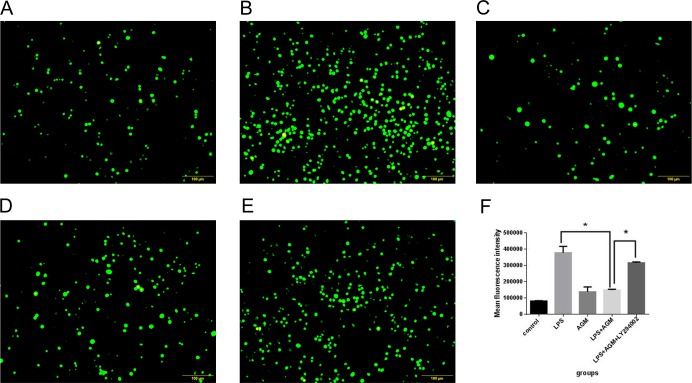
Agmatine inhibits LPS-induced ROS generation via the PI3K/Akt pathway. (A) RAW264.7 cells were cultured in RPMI 1640 for 24 h followed by incubation with the DCFH-DA probe (20 μmol/L) at 37°C for 30 min. (B) RAW264.7 cells were stimulated with 10 μg/mL LPS for 24 h followed by incubation with the DCFH-DA probe for 30 min. (C) RAW264.7 cells were treated with 1 mM agmatine for 24 h. (D) RAW264.7 cells were incubated with 10 μg/mL LPS and 1 mM agmatine added 30 min later for 24 h, and then ROS was detected with the DCFH-DA probe for 30 min. (E) RAW264.7 cells were pretreated for 1 h with 10 μM LY294002, and then incubated with 10 μg/mL LPS and 1 mM agmatine added 30 min later for 24 h. (F) ROS production was analyzed by flow cytometry. Results are means ± SEM of three independent experiments and were assessed by one-way ANOVA. * P < 0.05 and ** P < 0.01.

To further investigate the intracellular signaling pathways that are activated by agmatine to induce the nuclear translocation of Nrf2 and subsequent expression of HO-1, the PI3K/Akt pathway was investigated, as this has been recently demonstrated to be an important upstream regulator of HO-1 expression. Western blot analyses were performed to detect the level of Akt phosphorylation. Our results showed that agmatine significantly enhanced Akt phosphorylation after 24 h treatment (Fig 5A); total Akt protein levels were unaffected by agmatine treatment. To further confirm the role of PI3K/Akt in the anti-oxidative effect of agmatine, PI3K inhibitor LY294002 (10 μM) was induced, and the results showed that the antioxidant effects of agmatine were abolished. Both Nrf2 activation and HO-1 production were significantly attenuated by LY294002 (Fig 5B–5D), and ROS generation was increased (Fig 6). Together, these data suggest that agmatine-induced Nrf2 activation is associated with PI3K/Akt signaling.

### Inhibiting HO-1 pathway significantly attenuated the antioxidant effect of agmatine on inflammatory macrophages

**Fig 7:**
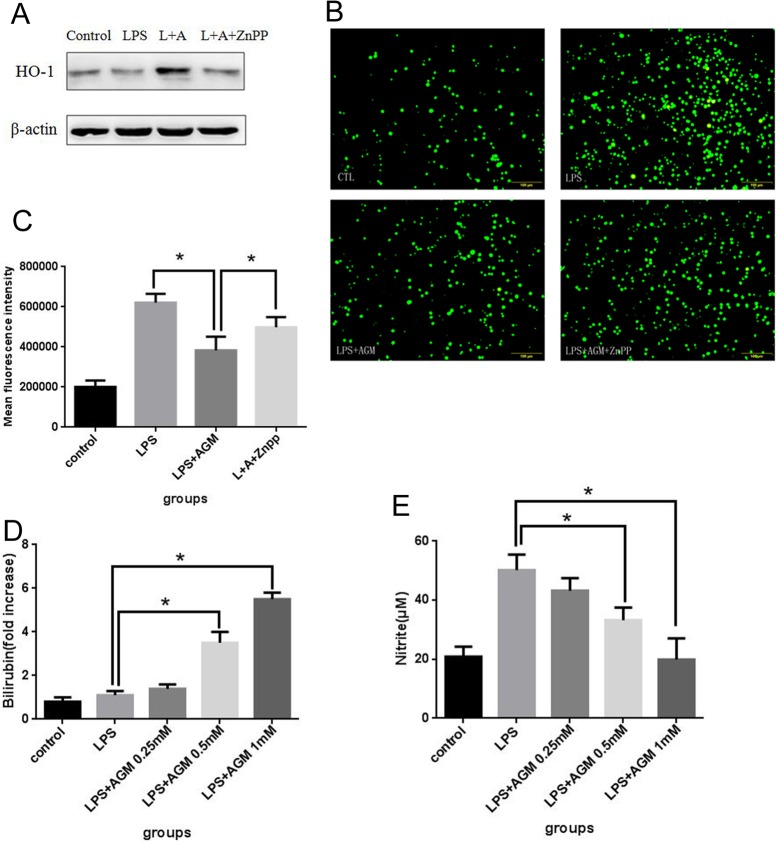
agmatine exhibits its antioxidant effects on inflammatory macrophages mainly through a HO-1-dependent manner. (A) Raw 264.7 cells were cultured as described in Fig 2 legend and were treated with ZnPP (1 μm) for 1 h before incubation with LPS (10 μg/mL) and agmatine (1 mM) added 30 min later for 24 h. HO-1 protein expression was analyzed by western blotting. (B and C) Cells were treated as described in A. ROS was labeled by DCFH-DA probe. ROS level was assessed by fluorescence microscopy (B) and flow cytometry (C). (D) Bilirubin accumulated over time was measured in the supernatant of Raw 264.7 cells 24h after exposure to increasing concentrations of agmatine (0.25, 0.5 and 1 mM) and is shown here as fold increase over the control.(E) Nitrite levels in the supernatant were evaluated using a colorimetric assay based on the Griess reaction. Data represent the mean ± SEM of 3 independent experiments per group and were assessed by one-way ANOVA. * P < 0.05 and ** P < 0.01 indicate significant differences compared with the control group.

Blocking experiments were conducted to reveal the importance of HO-1 pathway in the protective properties of agmatine.As shown in Fig 7B, agmatine suppressed LPS-induced ROS generation to a great degree; however, this suppression was significantly abolished when the cells were pretreated with HO-1 inhibitor ZnPP. These results suggested that agmatine exhibits its antioxidant effects on inflammatory macrophages mainly through a HO-1-dependent manner. In addition, numerous evidences showed that the final degradation products of heme catalyzed by HO-1, iron ions, carbon monoxide and bilirubin mediated the protective properties of antioxidant and anti-inflammation, so we verified if the up-regulation of HO-1 levels by agmatine was associated with a rise in HO-1-derived products. Since the generation of three components must be concomitant, we detected the levels of bilirubin in supernatant after treated with agmatine for 24 h. As shown in Fig 7D and 7E, the levels of bilirubin were not affected by LPS stimulation. But after treated with agmatine, bilirubin in the cell supernatant increased significantly in a concentration-dependent manner, accompanied by a reduction of nitrite production elicited by LPS.

### Membrane receptors are not required for the anti-oxidant effects of agmatine in RAW 264.7 cells

**Fig 8:**
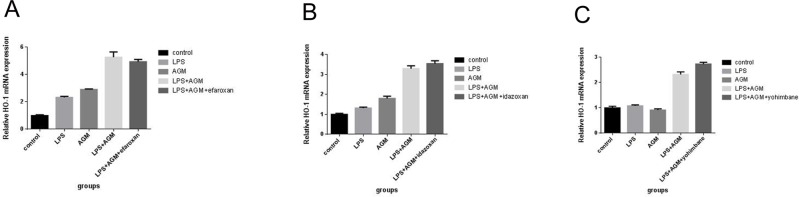
Membrane receptors contribute to the anti-oxidant effects of agmatine in RAW264.7 cells. RAW264.7 cells were treated with 10 μg/mL LPS for 30 min and then co-incubated with agmatine (1 mM) for 24 h in the presence of 100 μM efaroxan (EFA), 100 μM idazoxan (IDA) or 100 μM yohimbine (YOH). (A, B and C) HO-1 mRNA expression after intervention was analyzed by qPCR. None of these inhibitors blocked the protective effects of agmatine. Each column represents the mean ± SEM of three independent experiments. Statistical analysis was performed by one-way ANOVA.

To assess the membrane receptors were required for the anti-oxidant effect of agmatine, RAW 264.7 cells were pretreated with different inhibitors for 30 min prior to the addition of LPS. Three inhibitors: yohimbine, α2-adrenoceptor antagonist (100 μM), efaroxan, I1-imidazoline receptor antagonist (100 μM) and idazoxan, I2-imidazoline receptor antagonist (100μM) were used. However, none of these inhibitors blocked the protective effects of agmatine, as shown in Fig 8.

## Discussion

Agmatine has been reported to possess a variety of beneficial biological properties, including blood pressure regulation, antioxidant and anti-inflammatory activities [21, 22]. Moreover, it is known to protect against chronic neuropathic pain, depression and anxiety in the nervous system [23]. In terms of its underlying mechanism of action, previous reports have suggested that agmatine inhibits the production of NO by decreasing the levels of NOS-2 protein in macrophages, which suggests a molecular basis for the anti-inflammatory actions of agmatine [24]. Recently, several reports have shown that antioxidant property of agmatine is likely to be a crucial mechanism of its antidepressant-like effects [25]. However, it remains unclear whether the antioxidant property of agmatine is the relevant mechanism in LPS-activated macrophages in peripheral tissues. Moreover, few studies have investigated the therapeutic effect of agmatine *in vivo* or *in vitro*. Our results showed that 24 h agmatine treatment significantly inhibited the generation of ROS and NO in LPS-activated RAW 264.7 cells, suggesting that agmatine is capable of attenuating oxidative stress and counteracting ROS-induced oxidative damage. This therapeutic effect is more meaningful than the preventive effect of agmatine and may provide a treatment strategy for clinicians.

Macrophages are the primary responders to inflammation, and induce immune responses to endogenous and exogenous stimuli, playing a central role in host defense and immune regulation. Increasing evidences have shown that activated macrophages enhance oxygen depletion, which causes the overproduction of ROS [26, 27]. HO-1, an antioxidative enzyme regulated by Nrf2 activation, is essential for redox homeostasis by preventing the production of ROS [28, 29]. Moreover, further research has found that antioxidant signaling via the Nrf2/HO-1 pathway counteracts stimulated inflammatory responses in various macrophage subtypes such as microglia, alveolar and peritoneal macrophages [30–32]. Therefore, we hypothesized that agmatine inhibits the induction of ROS through agmatine-mediated Nrf2/HO-1 expression. Our results suggested that LPS did not stimulate the up-regulation of Nrf2 or HO-1 in RAW 264.7 cells. Agmatine treatment could increased nuclear Nrf2 level and HO-1 expression significantly. Therefore, we concluded that agmatine exhibited antioxidant activity via up-regulating Nrf2-mediated HO-1 expression. However, the levels of other antioxidative enzymes, NQO-1 and GCLc, were not up-regulated, which suggested that Nrf2 regulates the expression of downstream antioxidative genes in a complex and not fully understood mechanism. Although Nrf2 is known to be a crucial upstream mediator of ARE-dependent phase II enzyme expression, our data suggested that its activity was not general to all ARE-containing elements.

Among various antioxidative gene products, HO-1 has attracted great interest for oxidative susceptibility and antioxidative effectiveness. The protective action mediated by HO-1 against oxidative damages has been thought to result from multiple mechanisms. As a cytoprotective enzyme, HO-1 catalyzes the rate-limiting step in heme degradation and generates three final by-products including iron ions, bilirubin and CO. Bilirubin, generated by the reaction of biliverdin and biliverdin reductase, is a potent radical scavenger that eliminates lipid peroxide radicals. CO could attenuate the catalytic activity of some oxygenases which lead to endogenous generator of ROS and suppress excessive NO generation. In addition, some evidences have shown that the activity-lacking mutant HO-1 can also protects cells against oxidative stress. We hypothesized that the products of HO-1 enzymatic activity contribute to the antioxidant effect of agmatine. Our results showed that bilirubin increased significantly in a concentration-dependent manner after treated with agmatine therapeutically, accompanied by a reduction of nitrite production elicited by LPS. Besides, inhibiting HO-1 pathway significantly attenuated the antioxidant effects of agmatine. Therefore, we conclude that agmatine exhibits its antioxidant effects on inflammatory macrophages mainly through a HO-1-dependent manner, resulting from mechanisms closely linked with the biological properties of the products of HO-1 enzymatic activity.

PI3K/Akt, a multifunctional signaling pathway that is associated with cell proliferation, apoptosis and cellular defense, has been reported to regulate Nrf2 [33–35]. Crosstalk between the PI3K/Akt and Nrf2 signaling pathways is capable of protecting cells against inflammatory and oxidative damage [36]. Subsequently we investigated whether the PI3K/Akt pathway mediates agmatine-induced Nrf2 activation and HO-1 expression. The results showed that agmatine induced a significant augmentation of Akt phosphorylation with no changes in the total levels of Akt protein. Subsequently, LY294002, a specific PI3K/Akt inhibitor, was used to further reveal whether activation of the PI3K/Akt pathway is directly associated with Nrf2-mediated antioxidant response. As expected, inhibition of PI3K/Akt signaling with LY294002 significantly blocked the activation of Nrf2 and HO-1expression induced by agmatine. Furthermore, the findings also showed that the ROS scavenging capability of agmatine could be attenuated by LY294002 obviously. Therefore, we concluded that the PI3K/Akt pathway plays a vital role in the agmatine-mediated antioxidant response.

Agmatine activates several postsynaptic membrane receptors, including imidazoline receptors and α2-adrenergic receptors [37, 38]. Additionally, it inhibits membrane Ca^2+^ channels and blocks N-methyl-D-aspartate (NMDA) receptors [39]. Imidazoline receptors play important roles in cell proliferation, neuroprotection, inflammation and psychiatric disorders [40]. Three subtypes of imidazoline receptors have been identified, and all have been reported to contain a binding site for agmatine [41, 42]. A previous report suggested that agmatine exerts its neuroprotective effects through a mechanism that involves Nrf2 induction via α2-adrenergic and 5-HT2A receptors in HT22 cells. There is a vast literature demonstrating that agmatine can activate biological cascades through α2-adrenergic receptors [43, 44]; therefore, we hypothesized that α2-adrenergic or imidazoline receptors were the membrane receptors targeted by agmatine and involved in its antioxidative effects. Yohimbine (an α2-adrenoceptor antagonist), efaroxan (an I1-imidazoline receptor antagonist) and idazoxan (an I2-imidazoline receptor antagonist) were tested for the ability to inhibit the effects of agmatine. However, our results showed that none of them attenuated the antioxidant response mediated by agmatine, suggesting that agmatine may afford the reduction of oxidative stress through other receptors in macrophages.

In conclusion, our study identified that agmatine may attenuate LPS-stimulated oxidative stress in macrophages through a mechanism that involves activating Nrf2 and increasing HO-1 expression via PI3K/Akt pathway. Furthermore,the products of HO-1 enzymatic activity contribute to the antioxidant effect of agmatine. Although the membrane receptors required for its antioxidant properties were not confirmed, our findings provide new insights into the potential therapeutic applications of agmatine for oxidative stress. As inflammation and oxidative stress are two biological events that are relatively independent but closely linked in many pathological processes, our findings may represent potential targets for the prevention and treatment of inflammatory diseases.
